# Genetic Biocontrol for Invasive Species

**DOI:** 10.3389/fbioe.2020.00452

**Published:** 2020-05-25

**Authors:** John L. Teem, Luke Alphey, Sarah Descamps, Matt P. Edgington, Owain Edwards, Neil Gemmell, Tim Harvey-Samuel, Rachel L. Melnick, Kevin P. Oh, Antoinette J. Piaggio, J. Royden Saah, Dan Schill, Paul Thomas, Trevor Smith, Andrew Roberts

**Affiliations:** ^1^ILSI Research Foundation, Washington, DC, United States; ^2^The Pirbright Institute, Woking, United Kingdom; ^3^Centre for Environmental Sciences, Hasselt University, Diepenbeek, Belgium; ^4^Commonwealth Scientific and Industrial Research Organisation (CSIRO), Wembley, WA, Australia; ^5^Department of Anatomy, School of Biomedical Sciences, University of Otago, Dunedin, New Zealand; ^6^National Wildlife Research Center, USDA/APHIS-Wildlife Services, Fort Collins, CO, United States; ^7^Island Conservation, Santa Cruz, CA, United States; ^8^Fisheries Management Solutions, Inc., Boise, ID, United States; ^9^School of Medicine, University of Adelaide, Adelaide, SA, Australia; ^10^Florida Department of Agriculture and Consumer Services, Gainesville, FL, United States

**Keywords:** invasive species, genetic biocontrol, gene drive, Trojan Female Technique, Trojan Y Chromosome

## Abstract

Invasive species are increasingly affecting agriculture, food, fisheries, and forestry resources throughout the world. As a result of global trade, invasive species are often introduced into new environments where they become established and cause harm to human health, agriculture, and the environment. Prevention of new introductions is a high priority for addressing the harm caused by invasive species, but unfortunately efforts to prevent new introductions do not address the economic harm that is presently manifested where invasive species have already become established. Genetic biocontrol can be defined as the release of organisms with genetic methods designed to disrupt the reproduction of invasive populations. While these methods offer the potential to control or even eradicate invasive species, there is a need to ensure that genetic biocontrol methods can be deployed in a way that minimizes potential harm to the environment. This review provides an overview of the state of genetic biocontrol, focusing on several approaches that were the subject of presentations at the Genetic Biocontrol for Invasive Species Workshop in Tarragona, Spain, March 31st, 2019, a workshop sponsored by the OECD’s Co-operative Research Program on Biological Resource Management for Sustainable Agricultural Systems. The review considers four different approaches to genetic biocontrol for invasive species; sterile-release, YY Males, Trojan Female Technique, and gene drive. The different approaches will be compared with respect to the efficiency each affords as a genetic biocontrol tool, the practical utility and cost/benefits associated with implementation of the approach, and the regulatory considerations that will need to be addressed for each. The opinions expressed and arguments employed in this publication are the sole responsibility of the authors and do not necessarily reflect those of the OECD or of the governments of its Member countries.

## Invasive Species

As global trade increases the transfer of goods and commodities around the world, it leads to the movement of species from their native ranges to new locations where they become invasive. According to United States Executive Order 13112 of February 3, 1999 (Invasive Species), invasive species are defined as “an alien species whose introduction does or is likely to cause economic or environmental harm or harm to human health” (Executive Order 13112, 1999). Whether or not a species becomes invasive depends on the characteristics of both its life history traits and the environment in which it is introduced. Not all new species introductions become invasive, however, species that become invasive in a new environment can have profound effects on industry, agriculture, and conservation lands within the new location where they become established (Paini et al., 2016).

Eradication of invasive species is often impractical once they have become established (Britton et al., 2011). In some cases, it is possible to contain invasive species at the site where the initial introduction occurred and eradicate the population before it has the opportunity to spread beyond the limits of the area where eradication measures are effective (Steck et al., 2019). In other cases, eradication is not considered an option and management plans are instead implemented as a means of preventing further spread. Most control efforts primarily focus on limiting the harm associated with the invasive species to an acceptable level. In many cases this may involve integrated pest management (IPM), an approach which utilizes multiple pest management tools in the hopes of obtaining a synergistic control effect (Kogan, 1998). Although the result of such an approach can be expensive and sometimes inefficient, there are few options to effectively limit the harm associated with invasive species.

## Genetic Biocontrol Strategies

Genetic biocontrol provides opportunities for the control and potential eradication of invasive species. The term “genetic biocontrol” refers to techniques that alter the genetic material of an organism to control invasive species in the environment. Some, but not all, of these techniques involve knowledge or manipulation of the genome. This includes utilization of naturally occurring genotypes; parasitic microbes that distort sex ratios; the use of traditional methods such as irradiation, hormonal sex reversal to generate sterile, or sexually incompatible genotypes; and the use of modern genetic engineering technologies. Because genetic biocontrol describes a wide variety of methods that take advantage of species biology in order to achieve control, it is important to note that genetic biocontrol is not a synonym for the use of genetically engineered organisms. Existing technologies that use naturally occurring genetic alleles, irradiated organisms, chromosomal segregation techniques, or endoparasitic bacteria (i.e., *Wolbachia*) constitute genetic biocontrol techniques that would not be considered genetic engineering.

This review provides an overview of the state of genetic biocontrol, focusing on several approaches that were the subject of presentations at the Genetic Biocontrol for Invasive Species Workshop in Tarragona, Spain, March 31st, 2019, an OECD Co-operative Research Programme sponsored conference. The review will highlight the range of genetic biocontrol options that are available (or are in development) for invasive species control, examining the attributes of four approaches; sterile-release, YY Males, Trojan Female Technique, and gene drive. The review compares techniques regarding the mechanism of control used in each method, and the regulatory hurdles that must be overcome for the genetic biocontrol methodology to be put into practice. Because gene drive is a fundamentally new approach to invasive species control that presents new possibilities for efficient suppression of invasive species populations (and may therefore pose new risk as well), research efforts to test containment for gene drives (both physical and genetic containment) are also reviewed. Lastly, the paper presents a review of classical biocontrol to provide context around ongoing practices for invasive species management and to highlight existing regulatory frameworks involving the release of a new organism into the environment that are likely to be relevant to the use of newer genetic biocontrol methods.

### Sterile Insect Technique (SIT) as a Reference Point for Considering Genetic Biocontrol

One of the earliest applications of genetic biocontrol involved irradiation of the screw worm *Cochliomyia hominivorax* (Coquerel) as a means of producing sterile individuals that could be generated in large numbers and distributed within a target population in order to suppress reproduction. The technique, known as Sterile Insect Technique (SIT) successfully eradicated screw worms from the southeastern United States (Knipling, 1955; Smith, 1963), and has since been employed for the control of a variety of other insects [e.g., the codling moth (*Cydia pomonella* (L.)) (Bloem et al., 2007)], the pink bollworm [*Pectinophora gossypiella* (Saunders) (Tabashnik et al., 2010)], and the painted apple moth [*Teia anartoides* (Walker) (Suckling et al., 2007)]. Preliminary work on vectors of human disease such as the yellow fever mosquito [*Aedes aegypti* (L.) (Alphey et al., 2010)] and Tsetse fly [*Glossina* spp.) (Vreysen et al., 2014] has been conducted, with more work needed to achieve effective control by sterile-release.

Insects are irradiated for SIT using a dose of gamma radiation sufficient to cause chromosomal breaks within the germ line. For some insect species, SIT does not substantially alter mating competitiveness. However, there are limitations to this technique in that some species have incomplete sterilization, causing a serious decrease in competitiveness and an insufficient sterile population, amongst other issues (Esteva and Mo Yang, 2005). If it is practical to do so, males are sorted from females to allow release of only sterile males into the environment. This leads to a more efficient suppression of the target population (up to 3–5-fold) than if both sexes are released together (Rendón et al., 2004; Alphey and Bonsall, 2018). Irradiated insects are then released into the target area in sufficient numbers to ensure that fertile individuals will have a high probability of encountering irradiated sterile individuals leading to unproductive mating.

Although SIT has been a successful approach to control some insect pests, there are disadvantages associated with its use. In order to be successful in suppressing a target population, an overwhelming number of irradiated organisms must be released into the environment, thus temporarily increasing the potential impacts caused by the pests. To produce the required numbers of sterile insects, a dedicated facility to rear and irradiate organisms for release is required, which can be costly to construct and operate. Some species are not suitable for sterile release because of limitations in rearing, thus the technique may be unsuitable for control of some species. The effects of radiation on insect mating competitiveness can also be a disadvantage, requiring that even greater numbers of irradiated insects are released in order to compensate for reduced mating efficiency.

**FIGURE 1:**
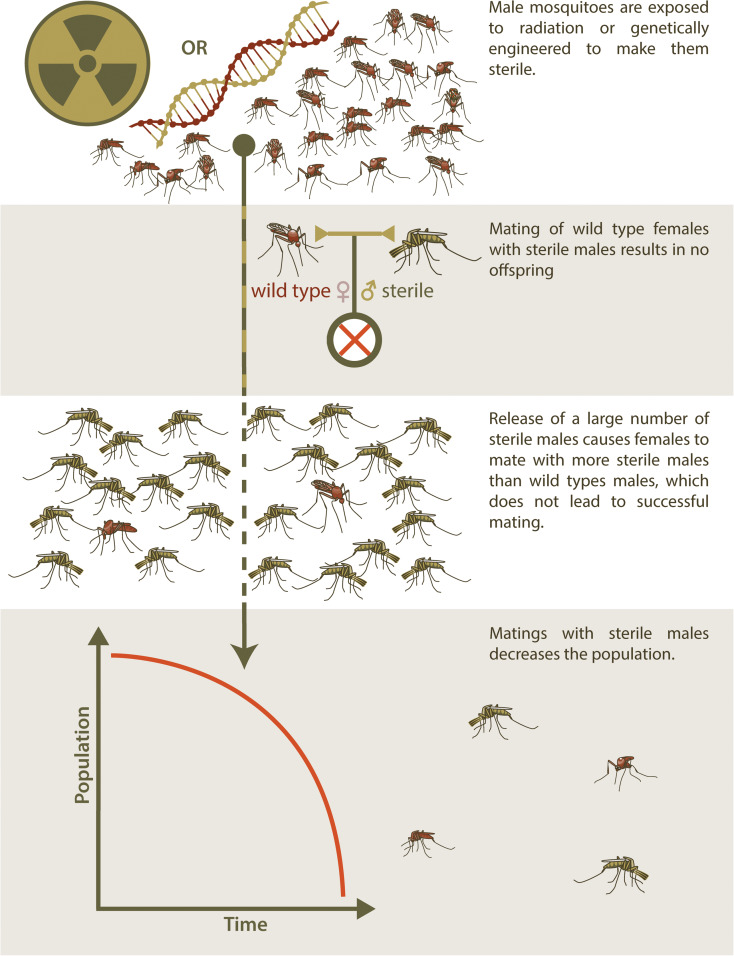
Sterile Insect Technique. The release of sterilized males for population control is used to manage populations of invasive species through a method known as Sterile Insect Technique (SIT). Initially, a seed colony is maintained, from which batches of eggs are taken and amplified for several generations to produce large release cohorts. In traditional SIT, males are exposed to radiation to induce sterility, but newer technologies have made it possible to genetically engineer sterile males and potentially avoid the fitness and mating efficiency costs that accompany irradiation. Mating between these sterile males and wild type females do not result in offspring. These sterile males must be released into the wild population in very large numbers, so that wild type females are more likely to mate with sterile males than wild type males, in order to effectively reduce the population of the invasive species over time.

As an alternative to SIT, genetic engineering has also been used to produce sterile insects for release to suppress a target population (Alphey and Bonsall, 2018). This technique has resulted in transgenic insects that are reproductively sterile as a result of a transgene that confers a dominant lethal phenotype to progeny that inherit it (RIDL, Release of Insects Carrying a Dominant Lethal) (Alphey, 2014). As with SIT, an overwhelming abundance of RIDL males must be released into a target population in order to ensure that wildtype females have only unproductive matings with RIDL partners and population suppression ensues (Figure 1). RIDL is thus an improvement over SIT with respect to practical aspects of producing insects with higher mating competitiveness but shares the same disadvantage of SIT in that many insects must be reared and released. This intrinsic disadvantage of sterile-release is also relevant to the use of this method to control non-arthropod invasive species.

Invasive bullfrogs [*Lithobates catesbeianus* (Shaw)] in Europe are not amenable to mass sterilization by radiation, therefore require an alternative strategy to produce sterile individuals for sterile-release. As demonstrated in a recent pilot study, induced triploidy can reliably produce sterile bullfrogs in sufficient numbers to eradicate a small target population under containment conditions (Descamps and De Vocht, 2017). Induced triploidy is a technique which disrupts meiosis by chemical, mechanism, or thermal methods, resulting in eggs or sperm containing three sets of chromosomes. Triploidy in reproductive cells results in abnormal development that can abort the reproductive process. However, future work on sterile-release efforts requires construction of a dedicated facility to rear sterile triploid bullfrogs in sufficient numbers for release. As adult bullfrogs can exceed 30 cm in length, the facility will need to be quite large and require substantially greater resources for care and feeding than insects. A further concern is that addition of an abundance of sterile bullfrogs to a target population will cause environmental harm. Such a program will thus require some form of manual wild bullfrog removal in an integrated pest management program to mitigate the harm associated with the introduction of additional sterile individuals. Such an approach melding population suppression and the addition of sterile individuals has been attempted with another invasive vertebrate, the Sea Lamprey in the St. Mary’s River, a tributary of the Laurentia Great Lakes (Bravener and Twohey, 2016). Further harm resulting from the introduced sterile bullfrogs might be reduced if sterile individuals are added at a very early stage of development so that the sterile individuals have a smaller impact as competitors for food with the native aquatic organisms higher in the food chain.

### Trade-Offs Between Efficiency, Control, and Uncertainty of SIT

Although sterile-release has been a very successful approach for the control of pest insects, radiation-induced sterility of invasive species for sterile-release has limited applicability as a genetic biocontrol strategy for most invasive species. The approach may be restricted to a subset of arthropod invasive species; those that can be reared, irradiated, and distributed without substantial negative effects on the viability and mating effectiveness of the released insects. For species not amenable to radiation-induced sterilization, other genetic approaches to sterility (e.g., induced triploidy for fish and amphibians) or the Trojan Female Technique (Gemmell et al., 2013) may offer an alternative approach to radiation for the production of sterile individuals. For insects, RIDL provides an alternative to SIT and can be applied to a variety of invasive insect species affecting agriculture (Ant et al., 2012; Leftwich et al., 2014; Alphey and Bonsall, 2018). However, the principal limitation in using sterile-release as a general approach for invasive species control is the problem of limiting the harm associated with the large number of sterile individuals required to suppress the fertility of the target population. In some cases, harm is specific to one sex only and can be avoided by appropriately sorting and releasing only sterile individuals of one sex, however, in many cases the harm is associated with both sexes. In these cases, an integrated pest management approach (i.e., removing fertile individuals from the target population and replacing them with sterile individuals) may be feasible as a means of augmenting the efficacy of a sterile-release approach without increasing the harm imposed by the released sterile individuals. However, IPM may not be feasible for many invasive species, especially those which are widely distributed over a large geographic area. The inefficiency of sterile-release is thus a barrier for its use to eradicate or control most invasive species.

Although the method of sterile-release may offer some utility for reducing or eradicating small populations of invasive species, more efficient genetic biocontrol methods are currently being explored that do not require the production of large numbers of individuals for release into the environment, and are not limited with respect to the size of the target population that can be targeted. Three of those approaches; YY Males, Trojan Female Technique, and gene drive; are considered in the following sections.

### YY Males (Trojan Y Chromosome)

**FIGURE 2:**
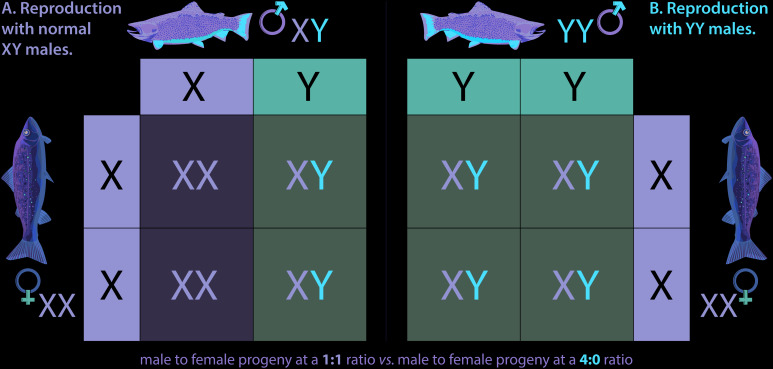
YY Males. Inheritance of sex chromosomes in Brook Trout **(A)** follows typical Mendelian patterns. Mating between a wildtype male (XY) and wild type female (XX) results in a 1:1 ratio of female (XX) to male (XY) fish in the offspring. When a YY male Brook Trout mates with a wild type XX female **(B)**, the only potential resulting genotype is male (XY), meaning all offspring are males. Continued introduction of YY Males in adequate numbers theoretically results in eradication of invasive Brook Trout populations over time.

Hamilton (1967) is credited with proposing that an undesired population could be eliminated by shifting the sex ratio completely to a single sex. The idea that such an anthropomorphic sex ratio shift might be accomplished by aquaculture-induced sex reversal in fish first occurred to John Teem who hypothesized that sex reversal in a captive broodstock via use of exogenous sex hormones could be used to produce a genetically all-YY Male broodstock whose progeny could be released into an undesired population (Mills, 2009). An application of this concept, termed the Trojan Y Chromosome (TYC) approach was formally explored first in a mathematical model evaluating the potential of the method for eradicating an invasive Nile Tilapia *Oreochromis niloticus* (L.) population (Gutierrez and Teem, 2006). In the TYC eradication approach, feminized, or egg-producing fish with two Y chromosomes are produced by commercial aquaculture practices involving selective breeding and sex-reversal by hormone treatment. These fish are then introduced into a target invasive fish population where they mate with normal males, giving rise to all male progeny, half of which will be sperm producing YY Males, further speeding the extirpation process (Teem and Gutiérrez, 2010). A variant of this original concept is to release sperm producing YY Males that would breed with wild females, resulting in all XY progeny (Figure 2). This approach is expected to be less efficient but also eradicated modeled invasive populations *in silico*, and was suggested to be more practical (Parshad, 2011), presumably because it would require the feminization of far fewer fish. Regardless of the type of YY Males being released, i.e., egg or sperm-producing males increase in the population over time at the expense of females until females are eventually eliminated, causing the population to collapse.

The development of a Trojan Y Chromosome broodstock for use in field studies as biocontrol agents was first undertaken for the Brook Trout (*Salvelinus fontinalis* (Mitchill), by the Idaho Department of Fish and Game (IDFG) in November 2008 (Schill et al., 2016). IDFG utilized the indirect broodstock development approach (Beardmore et al., 2001) and the use of a sex marker, PIT-tagging, and other production methods to develop a YY broodstock capable of producing large numbers of sperm-producing YY broodstock for field release in only three generations (Schill et al., 2016). In reporting their findings, the IDFG investigators preferred the use of the term “YY Males” over the previously used TYC label because it was more readily understood by the general public and decision-makers.

Having created the YY Male Brook Trout broodstock program in Idaho, population simulations were needed to provide guidance for field experiments and identify a range of likely stocking densities. The two most important predictions from the modeling exercises were the number of YY Male fish needed for release and the stocking duration in years to eradicate a target population (Gutierrez and Teem, 2006; Cotton and Wedekind, 2007; Stelkens and Wedekind, 2010). In addition, prior to 2016, all published TYC simulation authors (Gutierrez and Teem, 2006; Teem and Gutiérrez, 2010; Parshad, 2011; Parshad et al., 2013) had opted to evaluate only the addition of YY fish to invasive populations. None had evaluated other concurrent manual removal programs (hereafter suppression) as part of an integrated pest management program. Modeling of Brook Trout data from Idaho suggested that such a dual pronged IPM program would result in population extirpation within 2 to 4 years, assuming good YY Male fitness, and 5–15 years when YY Male fitness was only 20% that of wild males (Schill et al., 2017). Because stocking of YY Male fingerlings and manual suppression can readily be conducted at levels assumed in many of the simulations predicting complete eradication, Schill et al. (2017) recommend full-scale field testing of YY Male stocking in both streams and lakes within an IPM program that includes manual suppression.

Concurrent with the modeling exercises, Kennedy et al. (2017) conducted a pilot study to determine if stocked YY Male Brook Trout can survive, emulate the spawn timing of wild fish, reproduce with wild fish, and produce only XY males. YY Male Brook Trout were evenly dispersed in each of four pilot study streams in a single year and comprised an average of 3.1% of adult Brook Trout at spawning time several months later. Subsequent genetic assignment testing of Age 0 Brook Trout fry demonstrated that an average of 3.7% of fry collected the following summer were the progeny of YY Males and all were XY males, confirming that stocked YY Male fish can survive and spawn successfully with wild females and produce all-male progeny (Kennedy et al., 2018). Based on the positive pilot study results, IDFG subsequently expanded YY Male research efforts to full-scale field evaluations involving 13 waters including six alpine lakes and seven streams. The initial results of this research effort are just beginning to be documented.

The YY Male eradication technique offers an approach to eradicate invasive fish that does not use genetic engineering and is currently the only genetic biocontrol utilized in the United States. This technique is currently supported by fisheries resources managers in several western United States. If successful, it may provide a model for the development of other types of genetic biocontrol that similarly avoid the use of transgenics and are potentially applicable to a large number of invasive species.

### Trojan Female Technique

**FIGURE 3:**
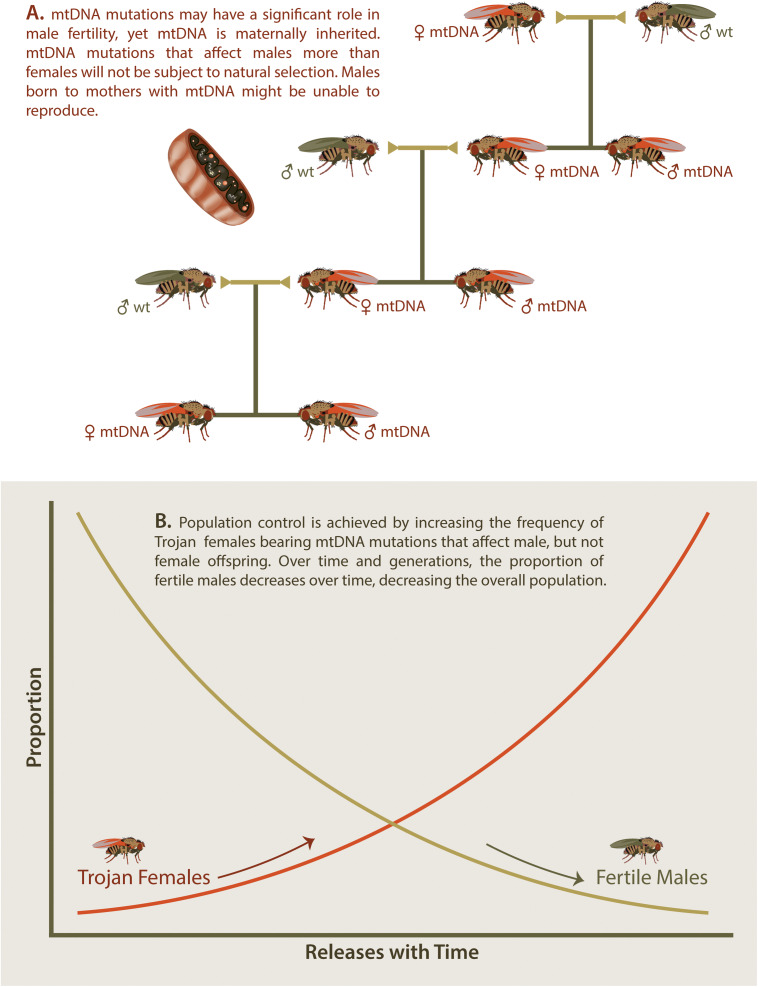
Trojan Y Females. Several mitochondrial DNA (mtDNA) mutations have now been identified that cause significantly reduced male fertility while having no effect on female fertility **(A)**. If females carrying such mtDNA mutations are introduced into wt populations, then male offspring will demonstrate reduced fertility while female offspring will continue to pass on the mtDNA to future generations. **(B)** Introduction of Trojan females increases the portion of Trojan females over time and leads to a concurrent decrease in fertile males over time. This decrease in fertile males will cause an overall decrease in the targeted invasive species in a similar manner to that observed with sterile insects.

The Trojan Female Technique, or TFT, is a novel twist on the sterile insect or sterile male approach. In the TFT, sustained population control is achieved through the steady release of “Trojan females” that carry mitochondrial DNA (mtDNA) mutations that cause reductions to male, but not female fertility (Gemmell et al., 2013; Figure 3A).

The TFT concept is enabled because of an evolutionary loophole that is common to most eukaryotic life. mtDNA is overwhelmingly inherited maternally thus mtDNA mutations that affect only males will not be subject to natural selection (Frank and Hurst, 1996). Theory predicts that such mutations can reach high frequencies in natural populations potentially impacting on their viability (Gemmell and Allendorf, 2001); an idea termed “Mother’s Curse” (Gemmell et al., 2004). If individuals carrying such naturally occurring mutations could be identified and cultivated, then the release of females carrying these mutations could, at least in theory, achieve self-perpetuating population control (Gemmell et al., 2013).

A variety of naturally occurring mtDNA mutations that reduce male, but not female, fertility have now been identified in fruit fly (Xu et al., 2008; Clancy et al., 2011; Yee et al., 2013; Patel et al., 2016), mouse (Trifunovic et al., 2004; Nakada et al., 2006), and European hare (Smith et al., 2010). The existence of these mutations in other species has not yet been extensively investigated. Given the ubiquity and conservation of mtDNA, it seems likely that these mutations occur in other species.

Modeling studies suggest that the TFT has the potential to achieve pest control under a wide range of conditions (Gemmell et al., 2013). Single large releases (10% of the population) and relatively few small repeat releases (1% of the population) of Trojan females both provided effective and persistent control within relatively few generations (Figure 3B). Although greatest efficacy was predicted for high-turnover species, the additive nature of multiple releases made the TFT applicable to the full range of life histories modeled. TFT mutations became increasingly less effective when males carrying Trojan mtDNA were only partially infertile, having lower fitness to wildtype males. Multiple female matings also reduced the effectiveness of the TFT (Gemmell et al., 2013).

Recent work in fruit flies (*Drosophila melanogaster*) supports the view that the TFT can reduce populations (Wolff et al., 2016). However, the level of population suppression achieved was modest, 8% across 10 generations, thus the TFT would need to have a stronger effect to have utility in the field. The search for TFT mutations that have stronger effects continues but is limited by the standing genetic variation in the populations that are being screened and remains time consuming. Thus, while one of the original strengths of the TFT approach is that it uses naturally occurring mtDNA variants (Gemmell et al., 2013) and, thus does not involve genetic engineering, directly or indirectly engineering one or multiple mutations into the mtDNA may enable more rapid discovery of TFT mutations.

Directly engineering mutations into mtDNA is far from trivial (Gammage et al., 2018a). Some researchers have reported success in modifying a series of nucleases to work efficiently in the mitochondria to cut and thus eliminate a defective mtDNA copy (Gammage et al., 2018b). However, the general inability to import nucleic acids into mitochondria severely limits the prospect of more direct manipulation using CRISPR based gene editing to introduce novel genetic variants – particularly beyond lower metazoans (Gammage et al., 2018b).

An alternative approach is to generate lines of animals that have defects in the polymerase responsible for the replication of mtDNA to rapidly develop and explore new mtDNA variants. Mice genetically engineered to express a proof-reading-deficient version of PolgA, the nucleus-encoded catalytic subunit of mtDNA polymerase, show a heightened incidence of *de novo* mtDNA mutation (Trifunovic et al., 2004). These mice have successfully been used to investigate the role of mtDNA in longevity (Vermulst et al., 2008) and disease, and may be a means through which novel mtDNA mutations can be generated and subsequently explored for male specific effects on fitness. Recently, 12 new mouse mitolines were established using PolgA founders and are currently assessing the effects of the mtDNA mutations we generated on fertility (Gemmell et al. unpublished). A similar experiment is now underway in fruit flies (Kauppila et al., 2018). However, this process relies on random mutation which is much less efficient than targeted gene editing approaches.

A potential middle ground approach is to directly identify mitochondria carrying desired mutations *in vitro*, and then transfer these mitochondria directly into developing zygotes (Nakada et al., 2006) or capture these using backcrossing experiments (Yu et al., 2009; Tourmente et al., 2017). Through such an approach, Nakada et al. (2006) developed a transmitochondrial mouse model (mito-mice) that carried wild-type mtDNA and a mutant mtDNA with a pathogenic 4,696 bp deletion (ΔmtDNA). Refinements on this approach, wherein mtDNA variants are generated using classic molecular biology or recent synthetic biology approaches, could establish a framework to target specific sites in the mtDNA and test their effects on male and female fertility, and ultimately their potential application in the TFT.

Although the TFT shows promise as a species-specific, reversible, and humane form of population control that has more support from the public than many alternative technologies (Gemmell et al., 2013), there are substantial hurdles to be overcome. Foremost among these is that the effects observed via empirical experimentation are weak, such as only 8% fruit fly population reduction across 10 generations (Wolff et al., 2016) and has yet only modest effects observed on mtDNA type on mouse fertility (Tourmente et al., 2017).

## Compromising Efficiency to Gain Control

These methods, YY Males and TFT, are likely more efficient than sterile release, requiring substantially fewer Trojan individuals to be introduced into the environment in order to effect a change on the target population. Each strategy also provides natural resource managers with some measure of predictability and control in the eradication process, a feature that may be lacking in other approaches (e.g., gene drives). Both strategies require that a steady influx of Trojan individuals are added to a target population to cause eradication over time. However, by ceasing the addition of Trojan individuals, eradication efforts can be terminated. Having a means of terminating an eradication program is a feature that is important to natural resource managers as it reduces the risk of making mistakes that permanently change the population. Neither method involves the introduction of transgenic organisms or GMO’s into the environment (Cotton and Wedekind, 2009; McNair et al., 2015), which will likely be viewed as advantageous by resource managers. In contrast to TFT, the YY Male technology has been developed to the point of practical application and field testing for Brook Trout is ongoing in Idaho and three other western United States states including Oregon, Washington, and New Mexico. The TFT genetic biocontrol has not yet been developed sufficiently to allow practical application against invasive species. In theory, it should be broadly applicable to a variety of invasive species provided that the mitochondrial genome in the organism can be engineered. Unfortunately, genetic engineering of mitochondrial genomes is currently impractical, so the future benefit of the TFT strategy for invasive species genetic biocontrol has yet to be realized. More research is needed in mitochondrial genome engineering to determine if this non-transgenic approach can be applied more broadly to any invasive species other than fruit flies.

### Gene Drive

Gene drives are genetic elements with biased inheritance and have considerable potential for suppression of target pest populations (Burt, 2003; Sinkins and Gould, 2006). While naturally-occurring gene drives have been identified (e.g., T allele in *Mus musculus* L.), the recent advent of CRISPR/Cas9 gene editing technology has enabled generation of synthetic gene drives that in theory could be adapted for use in any sexually reproducing species (Esvelt et al., 2014). To date, most synthetic gene drive development has been performed in insect species including the experimental model *Drosophila melanogaster* Meigen (Gantz and Bier, 2015) and the malarial vectors *Anopheles stephensi* Liston (Gantz et al., 2015) and *Anopheles gambiae* Giles (Hammond et al., 2016, 2017). The relative success of these studies has generated considerable excitement in the conservation technology community and gene drives have been proposed as a “silver bullet” for eradication of invasive mammalian pests. However, despite their potential, efficient CRISPR gene drives have yet to be developed any vertebrate species outside of cage experiments.

**FIGURE 4:**
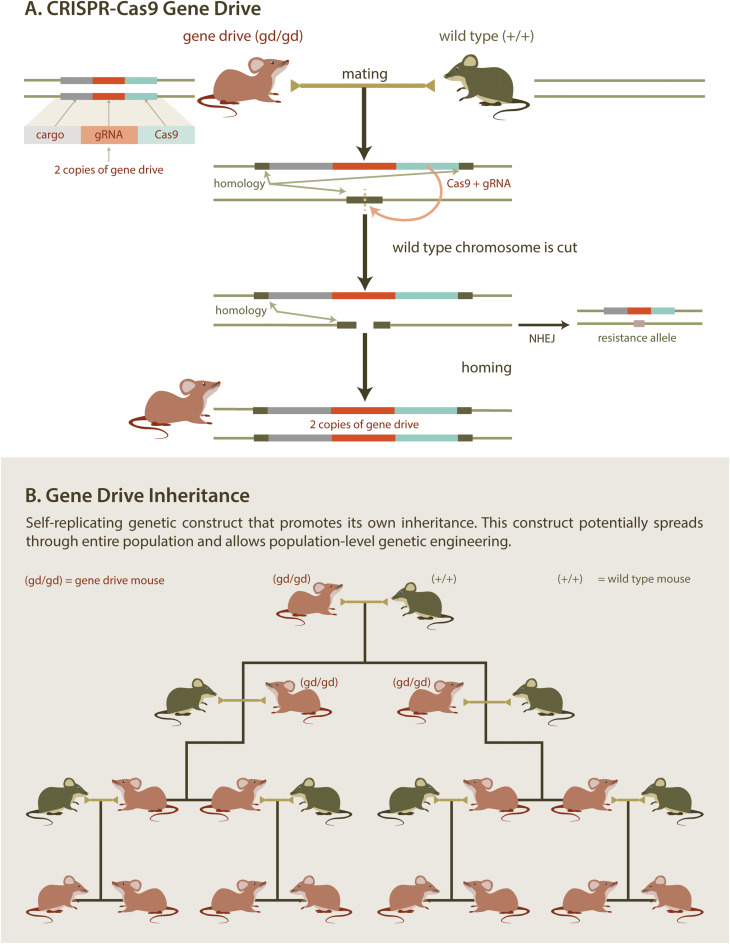
Gene Drive. CRISPR/Cas gene editing technology provides a practical new method to introduce genetic elements that bias inheritance. Mating mice containing a self-replicating gene drive element with wild type mice **(A)** results in offspring with two copies of the gene drive element. If the drive mechanism is efficient **(B)**, this allows for a desirable trait to be spread through an entire population. Desirable traits could include genes designed to reduce populations by, for example, skewing sex ratios.

At the molecular level, a synthetic gene drive consists of an expression cassette encoding a site-specific endonuclease (e.g., the CRISPR/Cas9 system). Importantly, this cassette is inserted into a chromosome at the genomic site that is cut by the endonuclease. Once the cassette has been integrated, the chromosome becomes immune to cleavage. Thus, a cell that is heterozygous for a gene drive cassette contains one allele that is susceptible to digestion by the endonuclease [the wild type (WT) allele] and one allele that is not (the gene drive allele). Expression of the gene drive endonuclease in a heterozygous cell will generate a double stranded break in the WT allele (Figure 4A). Repair of the double stranded break by homologous recombination (using the gene drive allele as a repair template) results in conversion of the WT allele to a gene drive allele, in a process termed “homing,” which renders the cell homozygous for the gene drive allele. Homing can be restricted to the gamete (egg/sperm) precursors resulting in selective homozygosity in the germline (i.e., the somatic cells remain heterozygous). Alternatively, homing can be directed to occur in the zygote (one-cell embryo). The homing event will ensure that the gene drive allele will be present in all gametes and will be passed on to all progeny. Over several generations, gene drives can spread rapidly through a given population (Figure 4B). While maximum gene drive spread occurs with 100% transmission, any increase above Mendelian (50%) transmission can still promote gene drive propagation throughout the entire population. Remarkably, gene drive transmission in mosquitos can be as high as 99.7%, indicating that CRISPR-mediated homing can be very efficient in insects (Gantz and Bier, 2015; Gantz et al., 2015; Hammond et al., 2016). However, gene drives with potential for field deployment are yet to be developed in rodents.

Other novel gene drive strategies based on innovative applications of CRISPR/Cas9 genome editing include the Y-CHOPE (Y-Chromosome deletion using Orthogonal Programmable Endonucleases) strategy (Prowse et al., 2019). This approach utilizes a standard homing cassette that also incorporates a programmable endonuclease that “shreds” the Y chromosome, thereby converting XY males into fertile XO females. The “shredding” of the Y chromosome using Cas9- or Cas12a-gRNA complexes that target repeat sequences on the Y chromosome has been demonstrated in embryonic stem cells (Adikusuma et al., 2017; Prowse et al., 2019). *In silico* modeling demonstrated that a Y-CHOPE gene drive can eradicate a pest vertebrate population. However, simulations indicate that, for polygynous species such as mice, Y-shredding efficiencies must be greater than ∼90% to produce high probabilities of eradication success (Prowse et al., 2019). Y-CHOPE may provide a useful alternative to the homozygotic XX sterility and homozygotic embryonic non-viability drives described above.

*In silico* modeling indicates that gene drives targeting female fertility genes and embryonic viability genes may be useful strategies for invasive mouse population suppression, and that a novel Y-shedding gene drive strategy has eradication potential (Prowse et al., 2019). Before any of these approaches can be considered for deployment, extensive engagement with stakeholders, regulators and the general community is essential. In addition, proof-of-concept studies in laboratory mice are required for development of field-ready tools. The time and effort required for technology development in mice should not be underestimated. It will likely be several years before the true potential of CRISPR gene drive technology in rodent can be fully appreciated. To date only a single paper has been published on gene drive homing in mice (Grunwald et al., 2019), and considerable optimization is required before this technology can seriously be considered for trials testing the ability to control mice populations. Non-homing approaches for rodent management such as X-shredder (McFarlane et al., 2018) and Cleave and Rescue (Oberhofer et al., 2018) strategies are therefore also worth exploring for conservation objectives.

#### Containment of Gene Drives

##### Testing of gene drives on islands

Gene drives have been proposed as powerful tools for controlling pest populations yet remain controversial. Perhaps the greatest unique risk potentially associated with these technologies is spread beyond the pest population which is being targeted (termed “transgene escape”), possibly affecting non-target populations or species. For species which are not “global targets” (i.e., those where the entire global population is the target), appropriate measures should thus be taken to reduce this risk, if these technologies are to be trialed in the field (Harvey-Samuel et al., 2019).

Transgene escape can occur in one of two ways. The first of these occurs at the spatial level in which the gene drive could move into a non-target population of the target species, for example from an area where the species is an invasive pest, back into the native range of that species. This is termed “intra-specific transgene escape” (Harvey-Samuel et al., 2017). Secondly, the gene drive could move into a closely related species at the release site (inter-specific transgene escape). These characteristics are most easily satisfied by oceanic islands, but sufficiently isolated ecological islands may also be sufficient.

##### Intra-specific transgene escape

Regardless of their mechanism, all gene drive systems are vertically transmitted technologies requiring gene flow to spread. Therefore, the level of gene flow between target and non-target populations a critical parameter in determining whether a gene drive transgene will escape a particular target population. Intra-specific transgene escape must therefore involve migration of individuals from the release area to non-target populations and subsequent introgression of their genomes into those populations. If sufficiently isolated from non-target populations, islands (geographical isolation or genetic isolation) provide high levels of ecological gene drive containment. The degree of containment offered will generally grow with increasing geographic distance between two populations, increasing ecological inhospitality of the intervening area and decreasing size of the population at the release site. In summary, small target populations surrounded by large distances of inhospitable terrain (including ocean if the target is non-marine) are the least likely to escape. Of course, what is deemed to be a sufficiently small population, or a great enough distance will be highly dependent on the ecological characteristics of the target species such as its reproductive rate and dispersal abilities. Additionally, possible human-mediated dispersal must be considered and should be minimized by careful trial site selection, biocontainment, and biosecurity.

An empirical field example of ecological containment can be found in the trialing of *Wolbachia*-based gene drives spreading through *Aedes aegypti* mosquito populations in Australia (Hoffmann et al., 2011; O’Connor et al., 2012; Hoffmann and Broadhurst, 2016; Schmidt et al., 2017) where release sites of *Wolbachia* infected mosquitos were separated from ecologically hospitable areas by a relatively impassable barrier. The gene flow between the two populations was sufficiently restricted such that the drive would not spread effectively between *Wolbachia* infected and native populations. However, when release sites occurred within a larger contiguous population, gene flow occurred at a high enough rate for the gene drive to spread beyond the target site and invade the wider population.

Another characteristic of island populations that reduced inter-specific escape is their relatively low genetic diversity and, specifically, the high frequencies of fixed alleles arising from the founder effect and subsequent drift in small initial generations. This can be advantageous for licensing sequence-specific drives (e.g., those based on CRISPR technology) to that particular target population, if a sequence can be identified which is fixed in the target population but not in non-target populations. This “genetic gene drives containment” remains robust even if the targetable sequence is present in non-target populations (Sudweeks et al., 2019).

##### Inter-specific transgene escape

Another potential route for transgene escape is through hybridization between the target species and a closely related species at the release site, followed by introgression of gene drive alleles by hybrids back into the non-target species. As with intra-specific transgene escape, there are several factors which can aid in reducing inter-specific transgene escape at island site locations. First, for hybridization to occur, there must be a closely related species (most likely a congenic species) at the release site. The risk of inter-specific transgene escape can thus be drastically reduced by choosing locations which are devoid of species congenic with the target. In some cases, the nature of hybridization events and the mechanics of the drive may also preclude transgenic hybrids being formed, even if congeners are present. For example, if hybridization is unidirectional with regards to sex and does not involve target species males, then drive systems which convert genotypic females to phenotypic males (such as Y-drive) (Burt, 2003) will be limited to the target population.

Secondly, there are factors which can act cumulatively to limit or prevent the risk of a drive spreading even if it does enter a non-target species. For example, if hybridization events do occur, they must result in fertile and competitive individuals that are able to introgress the drive into the non-target species. If hybrids are competitive, but hybridization events are rare, it is possible that stochastic loss of the drive will occur prior to the hybrid individuals being able to introgress the transgene into the non-target species. Similarly, for drive designs which require a minimum population frequency in order to spread, (Marshall and Akbari, 2018) the rate of this introgression may fall below that necessary for invasion of the non-target species. Finally, if competitive hybrids can introgress gene drive alleles into a non-target species at a relatively high rate, the drive would need to remain functional in this foreign genome. Even if regulatory components utilized to build the drive were compatible with the foreign species, loci targeted by the drive would also need to be present. The degree to which even small changes at the target locus can impair the spread of drives through relatively homogenous lab populations suggests that this scenario will be relatively unlikely. The simple nature of island communities means that examples where no closely related species occur will be relatively frequent, and that surveys of these communities to assess the risk of inter-specific transgene escape will be relatively easy to complete.

Gene drive system development is underway in several pest vertebrates. If promising technologies are to be trialed in the open field, there are advantages to conducting these trials on islands – whether oceanic or purely ecological. Small, ecologically simple, geographically isolated and genetically distinct island locations can aid in reducing the risk of unintended movement of the drive system into a non-target population. Islands offer the additional advantage of potential genetic containment through the exploitation of locally-fixed alleles.

#### Genetic Containment of Gene Drives – Locally Fixed Alleles

In order to reduce the uncertainties associated with the spread of gene drives, modifications to gene drives have been suggested that eliminate their capacity to propagate in a self-sustaining fashion, and instead allow them to persist for only a limited duration or in a limited area such as suggested by the LFA approach (Sudweeks et al., 2019). Daisy-chain gene drives (Noble et al., 2019) have been suggested as methods to limit the duration and spread of a gene drive. These techniques would have particular relevance to gene drives targeting invasive species because they would further mitigate the risk of unintended spread of the gene drive back to the native population.

Invasive mice on islands, which cause ecological destruction to sensitive island ecosystems (Campbell et al., 2019), are a good model system for exploring genomic targets for gene drive that could be specific to that population. Classic population genetic theory of founder events, genetic drift, and allelic fixation are the basis of assuming that invasive mice on islands, would have “locally-fixed alleles” that could then be used as targets for gene drive gene editing. A newly founded population of commensal rodents that invade an island from a ship would eventually lead to an island population that would have different allele frequencies than found in the continental population from which they originated. Further, some alleles might be fixed, found in each member of the population, due to genetic drift. These may not be alleles that are not found in the original populations, but they would occur in a much higher frequency (up to 100%) than the source populations. Identifying and exploiting such “locally-fixed alleles” would make a gene drive effective specifically in that island population and thus not be a risk for accidental spread to other geographical regions.

Mathematical models can be used to assess the effectiveness of a gene drive associated with an island population of locally-fixed alleles. Further, they can be used to test the spread of a drive in a continental population where the islands locally-fixed alleles may occur in low frequency. For gene drives to be effective, the target allele must be fixed in the island population meaning they are found at 100% frequency on the island. It is important to test this event as the possibility exists of a gene drive mouse getting transported to the continent as humans have long managed to transport commensal rodents unintentionally. Mathematical models demonstrate that, for a gene drive associated with an island locally-fixed allele, escape of a drive-bearing individual to from an island that was introduced into a continental population with that allele in low frequency would cause only a temporary decline in the continental population with a subsequent rebound in population numbers (Sudweeks et al., 2019). This suggests that if locally-fixed alleles are identified in an invasive, mouse island population that they could indeed serve as a biologically limiting gene drive target. It should be noted that mathematical models by Noble et al. (2019) indicated that gene drive systems have a highly likelihood for invasiveness into wild population, so contained field trials could have unintended spread of gene drive to other populations.

To investigate the feasibility of the locally-fixed alleles strategy, authors of this paper KO and AP used previously published genomic data to characterize the frequency of such alleles in an invasive island mouse population and a putative continental source population. As a part of their survey of wild *Mus* populations, Harr et al. (2016) compiled a whole-genome resequencing dataset that included samples (*N* = 3) of *M. musculus helgolandicus*, a subspecies found only on the German island of Heligoland, where it evolved from anthropogenic introductions of *M. m. domesticus* (Babiker and Tautz, 2015). We contrasted genomic variation in these samples with *M. m. domesticus* (*N* = 8) collected near Cologne-Bonn, Germany (Pezer et al., 2015) to identify locally-fixed alleles and characterize population differentiation. After downloading the aligned sequence files^1^ we first estimated genome-wide diversity as expected single-nucleotide polymorphism heterozygosity (SNP-*H*_e_) (Fischer et al., 2017) based on over 135 million autosomal sites. Consistent with the expectations for an isolated island population subject to a population bottleneck, *M. m. helgolandicus* mice had substantially lower levels of allelic diversity (SNP-*H*_e_ = 0.078) compared to continental mice (SNP-*H*_e_ = 0.315) in this new work. To assess population genetic differentiation, we calculated the fixation index (*F*_ST_) using the *poolfstat* R package (Hivert et al., 2018). Estimates of genome-wide allelic variation confirmed substantial population genetic differentiation (mean *F*_ST_ = 0.136) despite a relative short time (ca. 400 years) since island colonization and likely in the presence of persistent gene flow (Babiker and Tautz, 2015), which broadly points at the role of founder effects in population divergence (Carson and Templeton, 1984). More importantly to the present discussion, these results suggest that island colonization can have genetic effects on rodent populations that may result in viable genomic targets for population-specific synthetic gene drives. In this case, we scanned the genomic datasets for polymorphisms that created functional Cas9 protospacer adjacenet motif (PAM) sites in island populations but were absent in continental mice. Previous work has suggested that a single mutation within the PAM site is sufficient to preclude Cas9 genome editing activity (Hsu et al., 2013). Our analysis found 6,499 functional Cas9 PAM sites in Heligoland mice that were at absent in contential mice. Of these, 2,915 occurred in intergenic regions, which is desireable due to the reduced likelihood of unanticipated phenotypic effects in mice bearing the gene drive cargo (Prowse et al., 2017). Thus, the results of this pilot study suggest that genome engineers may have ample targets around which a gene drive could be designed for population-specific use in island populations, though we note the preliminary nature of these findings due to the low sample size. Selection of optimal target(s) from this pool of candidates should maximize efficacy while minimizing risk. Existing bioinformatic toolkits (e.g., VARSCOT)^2^, leverage machine learning and genetic/epigenetic diversity to compare efficacy amongst targets and to assess the likelihood of off-target and non-target effects (Cameron et al., 2017).

The locally-fixed allele strategy depends critically on identifying sites that are completely fixed in the target population, as rare variants in the PAM site will essentially create resistance alleles to the gene drive. Future efforts to screen genomic variation in island populations should carefully consider sampling strategies that will afford the greatest confidence in allele frequency estimates. Notably, evidence suggests that experimental designs that pool population samples prior to sequencing (i.e., “pool-seq”) may provide greater precision in allele frequency estimates compared to deep sequencing of a small number of individuals (Rode et al., 2018). To enable this, an expanded statistical analysis pipeline for pool-seq data has been developed (PeSTo)^3^, improving the power and speed of the original pool-seq pipeline (Anand et al., 2016). With access to sufficient population genomic data (within and between populations, target and related non-target species), an analytical pipeline that both identifies and evaluates targets for efficacy and risk can be linked to models of in-field propagation to conduct “digital” risk assessments of genetic control technologies that have yet to be developed by genome engineers.

## Risk Assessment

### Risk Assessment for Classical Biocontrol

Both genetic biocontrol and classical biocontrol involve the introduction of a new organism into the environment. Classical biocontrol is the release of a natural enemy to manage an introduced invasive species. Classical biocontrol agents are released with the understanding that the newly introduced organism will become permanently established in the environment. Because biocontrol effects of an introduced biocontrol organism are permanent and cannot be reversed, risk assessments for classical biocontrol agents must carefully consider risks that the new organism may pose to the environment. It is thus worthwhile to examine the risk assessment strategies used for classical biocontrol organisms and consider this risk assessment framework as a possible model for genetic biocontrol organisms.

Historically, classical biocontrol has been successfully applied to manage invasive species all over the world (Messing and Wright, 2006). Host specificity is the primary concern when considering the introduction of a biocontrol agent (McEvoy, 1996). Modern biocontrol programs require extensive host specificity testing in a quarantine environment and non-native organisms cannot be released without oversight from a rigorous scientific and regulatory vetting system (USDA, 2017). In the United States, this petition for release is submitted to the United States Department of Agriculture, Animal and Plant Health Inspection Service (USDA-APHIS) Biocontrol Program, with oversight from the Technical Advisory Group for Biological Control of Weeds (TAG-BCAW), which includes Canada, Agriculture and Agri-Food Canada, Mexico SAGARPA-SENASIA-DGSV, and other United States governmental agencies as cooperating organizations (USDA, 2017), allowing collaboration across North America. This strategy of testing and oversight has led to hundreds of successful biocontrol releases (Coulson, 1992; Hajek et al., 2007; Cock et al., 2016). To harmonize the regulation of invertebrate biocontrol agents in Europe, the International Organization for Biological Control of Noxious Animals and Plants (IOBC) organized the national regulatory framework on invertebrate biocontrol agents from across Europe into one guidance document (Bigler et al., 2010). Additionally, the FAO IPPC has established guidelines for the release of biocontrol agents (FAO, 2017).

When successfully implemented, classical biocontrol is an environmentally safe and cost-effective alternative to chemical pest control (van Lenteren et al., 1997; Bale et al., 2008). New technologies are becoming available to increase the effectiveness of classical biocontrol, such as sterile insect technique and gene drives. These new modified biocontrol strategies are the next step in the very successful evolution of biocontrol over the last century. The risk assessment framework for the release of classical biocontrol agents has a proven track record and offer a model for the release of many genetic biocontrol agents.

### Risk Assessment for Genetic Biocontrol

In order to establish whether an organism created using genetic biocontrol would pose a risk to the environment, it is necessary to perform an environmental risk assessment, a systematic procedure for predicting the possible harm that a hazard may pose to human health or the environment. A well-established framework exists for assessing the risk associated with the introduction of genetically modified plants into the environment (Raybould, 2006, 2007; Andersson et al., 2010; Garcia-Alonso and Raybould, 2014) as well as for classical biocontrol discussed in the previous section. These same frameworks can also be used to assess the potential risks associated with the introduction of a genetic biocontrol organism. Any of the approaches are not a one-size-fits all solution and will need to be evaluated for the specific situation in which they are used.

For release of genetic biocontrol agents, the first step in risk assessment will be problem formulation, where entities of value within the environment (also known as protection goals) that could be harmed by a gene drive are identified. Potential pathways leading to harm of the protection goals by the gene drive are then hypothesized (resulting in a risk hypothesis) and experimental data is collected to either validate or disprove the risk hypothesis. If experimental data suggests that no harm is likely to occur, this would provide information to decision-makers that the environmental risk posed by the new organism is low. Alternatively, the experimental data could indicate that the risk of harm is high, leading decision-makers to prohibit release of the new organism (or seek mitigation measures to reduce the risk to an acceptable level).

When considering release of genetic biocontrol agents, the first risk that needs to be assessed is whether release of a large population of reared individuals will have an impact on the environment, a risk assessment process similar to that done with classical biocontrol agents. This risk assessment can be done following the current framework for release of classical biocontrol agents. Release of mass-reared organisms is well established and conducted as part of area-wide IPM programs, and there is a history utilizing genetic biocontrol, such as SIT and *Wolbachia* infected insects, as part of such strategies (Mumford, 2012). When the organism is genetically modified, the environmental risk assessment should be done on a case-by-case basis. However, the risk assessment framework used for the release of mass reared insects and invertebrates helps to ensure that the appropriate science-based evidence is provided for the environmental risk assessment.

Gene drives may present a challenge for risk assessment in several ways. First, it will be necessary to clearly define what constitutes the “harm” that a gene drive poses to a protection goal in the environment. Spread of a trait within the environment may not itself constitute actual harm, but instead simply represents an event. Risk assessors will thus need to make a clear distinction between what constitutes harm and what does not. Another challenge for risk assessment of gene drives will involve testing risk hypotheses with experimental data. Trialing of gene drives will require a very high level of containment (ideally both physical containment and genetic containment). Although physical and genetic containment of gene drive mice on islands seems feasible, it may be difficult to find appropriate test conditions for gene drives constructed in other species. Mathematical modeling may therefore be an important means of providing data to inform risk assessments for gene drives for some invasive species. It should be noted that the regulatory framework noted above for classical biocontrol may not apply to genetic biocontrol and management of invasive fish and wildlife. In particular, in the United States, with the exception of migratory waterfowl and ESA listed species, wildlife (whether native or invasive) in the United States is owned, protected and managed by each of the states (Smith, 2011). This risk assessment framework that USDA-APHIS uses for classical biocontrol might not be relevant when considering genetic biocontrol for management of invasive vertebrate wildlife species in the United States.

## Public Perception of Genetic Biocontrol

Although scientific and regulatory hurdles exist for the practical use of genetic biocontrol to control invasive species, perhaps the greatest hurdle to be overcome will be public acceptance of the technology. Gaining public trust will also be an essential component in the development of new genetic biocontrol methods (and was identified in the workshop as the major barrier to implementation any genetic biocontrol).

The prospects for the development of genetic biocontrol to control invasive species will likely hinge on public perception of whether the use of such new technologies is sufficiently warranted to solve the problems being addressed. In a recent review of genetic control methods for invasive species, YY Male (TYC) was identified as the method least likely to generate public controversy (Thresher et al., 2014) though TFT and gene drives were not considered at that time. However, a recent Pew Research Center study (Funk and Hefferon, 2018) indicates public attitudes toward the use of genetic engineering on animals tend to be supportive if the technology is being applied to a major human health issue (e.g., preventing disease transmitted by mosquitoes). The public was less supportive of other uses involving the environment (e.g., increasing meat production for agriculture or recovering extinct species as a means of restoring biodiversity).

Whether the general public would consider the eradication of invasive species a problem that warrants the use of genetic engineering is yet undetermined. The review by Oliva et al. (2014) provides a historical context for how public perception has negatively impacted the implementation of classical SIT for vector management. A study on public perception of genetic biocontrol to control invasive fish found that a majority of people in the Great Lakes region were in favor of using genetic biocontrol to manage invasive fish populations, but recommended regulatory systems for the industry to help mitigate unintended consequences (Sharpe, 2014). In Mali, the public was open to the release of genetically modified mosquitoes to manage malaria, but wanted assurance that there would be no negative environmental or human health consequences (Marshall et al., 2010).

A landscape analysis on the use of gene drives in mice showed that the research community was concerned that gene drives would only receive public support if they could effectively eradicate the species, with a general concern that if implementation of gene drive were only partially successful, the public support for the strategy and even research on gene drive would be greatly diminished (Farooque et al., 2019). In a larger study of the public, the majority of the respondents were against gene editing of wildlife (71.3%), while 38.5% thought that using gene editing to control invasive species was not morally acceptable (Brossard et al., 2018). In general, there appears to be more support for genetic biocontrol of human disease vectors, but less support for management of wildlife. However, little was known about the perceptions of gene drive in agricultural systems. In a survey of over 1000 members of the public, Jones et al. (2019) the majority of respondent support the use of gene drive to control agricultural pests, if the mechanism limited spread.

This public perception is complicated by the fact that the science involved in invasive species eradication is often complex and is not currently well understood by or communicated to the general public. In a survey of the public on gene drive in agriculture, 85% of respondents were not aware of the existence of gene drive technology prior to receiving the survey (Jones et al., 2019). Public attitudes may therefore be determined, not by scientific debate regarding the risks of genetic biocontrol and mitigation strategies used to manage risk, but rather on the outcomes of initial field trials that allow the benefits of invasive species eradication. News articles involving eradication of invasive mice on an island by gene drive or eradication of invasive species could be how the public is introduced and educated on genetic biocontrol technology. In each case the term “genetic biocontrol” will be associated with whatever positive (and negative) outcomes arise from these efforts. The prospects for future applications of genetic biocontrol will thus likely depend on whether initial benefits achieved in these early studies are deemed commensurate with the perceived risk associated with the technology.

## Conclusion

Eradication of invasive species continues to be a challenge in a variety of ecosystems, ranging from heavily managed agricultural environments to wilderness areas. Tools for effective control are often inefficient and costly, making eradication of invasive species impractical once they have become established. While the potential exists to manage a range of pest with genetic biocontrol, hurdles remain with the implementation of these techniques. For some genetic biocontrol methods, one hurdle is the production of the large numbers of organisms that must be released into the environment. Gene drive, a genetic biocontrol method that is increasingly the focus of public attention, has the potential to spread without the need for sustained human intervention. This theoretically reduces the requirement for mass rearing and release of large numbers of organisms and would allow genetic biocontrol efforts to be applied to remote areas that are difficult to access or lack the infrastructure to support mass rearing efforts. Gene drive could also potentially be used to target invasive species that have become established over very large geographical areas (e.g., Asian carp in the Mississippi River, lionfish in the Caribbean, zebra mussels in the Great Lakes) (Harvey-Samuel et al., 2017). Although gene drive technology offers the potential for efficient, cost-effective, widely applicable genetic biocontrol control for invasive species, it has not yet been deployed in the environment and therefore also presents uncertainties with regard to both efficacy as well as potential unintended effects (Webber et al., 2015; National Academies of Sciences and Medicine, 2016).

Many genetic biocontrol methods, such as YY Males, require a steady influx of individuals with the biocontrol trait into a target population to cause eradication over time. While costly in terms of mass rearing requirements, these mechanisms offer the advantage of allowing for termination of releases if undesirable effects are observed. This can be very attractive to resource managers and to the general public. In contrast, at least some gene drive methods are intended to be self-sustaining. Consequently, the primary concern associated with the use of gene drive as a genetic biocontrol tool for invasive species is that it could spread to a non-target population causing unintended harm (Noble et al., 2018). Because of this and other reasons, the choice of any particular genetic biocontrol method will be informed by a variety of considerations, including the availability and amenability of technologies in the species of interest, the environment where the invasive species that is the subject of the control is present, public acceptance of the technology being applied and the cost versus benefit of deploying genetic biocontrol as part of a control program targeting any particular species.

**FIGURE 5:**
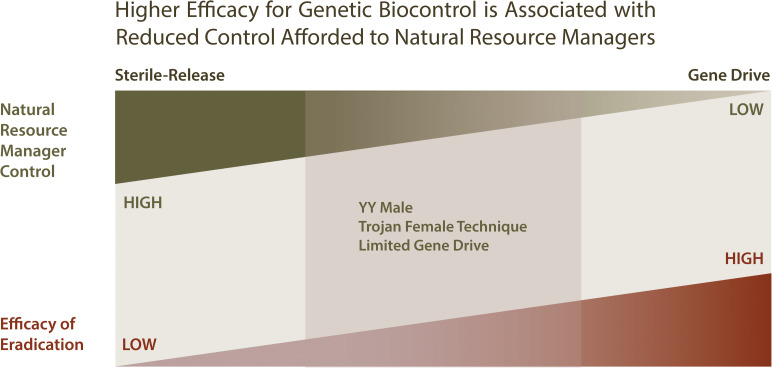
Comparing genetic biocontrol strategies with respect to efficiency and oversight control afforded to natural resource managers. When considering genetic biocontrol strategies, it can be helpful to consider the tradeoff between the efficiency of the genetic method, and the amount of control available to natural resource managers employing the strategy. Genetic biocontrol methods that are potentially the most efficient in eradicating invasive species include gene drives, which are self-sustaining and but thus require much initial from natural resource managers. Although gene drives are highly efficient for eradication, they provide natural resource managers with few options for managing the eradication process. In contrast, sterile-release methods provide an eradication process that is more easily managed, but less efficient, requiring the production and distribution of large numbers of sterile organisms into the environment in order to effect suppression. The YY Male and Limited Gene Drive genetic biocontrol strategies present alternative approaches that are intermediate with respect to efficiency and oversight control.

Sterile-release and gene drive represent two extremes within a continuum of genetic biocontrol approaches that vary with respect to efficiency vs. control afforded to natural resource managers, while YY Males and the Trojan Female Technique represent an intermediate in this continuum (see Figure 5). Sterile-release affords control natural resource managers with a large measure of control and little uncertainty with regard to unintended effects, but for a only a limited number of species and at a high cost with regard to infrastructure requirements. Although gene drive is likely to be more efficacious, require minimal infrastructure and address a greater number of invasive species, it does so at the cost of greater uncertainty of causing unintended environmental effects and providing limited options afforded to natural resource managers for oversight of control. As a compromise between these extremes, YY Male provides a method of genetic biocontrol with modest infrastructure requirements, modest genetic biocontrol efficacy that is applicable to a limited number of species, but with low uncertainty for unintended effects and providing a high measure of control to natural resource managers.

Together with certain technologies, including genetically engineered gene drive constructs or genetically engineered sterility systems genetic biocontrol options exist on a continuum and provide opportunities for the control and potential eradication of invasive species based on our knowledge of inheritance. While the techniques themselves are all unique, they fit under the broader category of genetic biocontrol, and all should be considered in the context of existing biocontrol and invasive species control programs. In considering how best to move forward with the development and deployment of genetic biocontrol methods, it is informative that public surveys on genetic biocontrol techniques reveal, perhaps unsurprisingly, that the general public simply are not educated on the techniques or even aware of them. This suggests that any program intending release of such organisms should also be associated with some form of educational campaign.

We suggest that resource managers, regulators and researchers should work together to ensure that several of these methods be deployed in the future to control invasive species while minimizing the impact on non-target species and the environment.

## Author’s Note

The findings and conclusions in this publication have not been formally disseminated by the U.S. Department of Agriculture and should not be construed to represent any agency determination or policy.

## Author Contributions

JT, LA, SD, ME, OE, NG, TH-S, RM, KO, AP, JS, DS, PT, TS, and AR all made significant contributions to the drafting of the manuscript. KO and AP conducted original data analysis for the manuscript. JT and AR organized the workshop. LA, NG, RM, AR, DS, and PT developed and created the figures. RM and AR lead the editorial efforts to develop the manuscript.

## Conflict of Interest

DS was employed by Fisheries Management Solutions, Inc. JS received travel support from the Outreach Network for Gene Drive Research Secretariat. The remaining authors declare that the research was conducted in the absence of any commercial or financial relationships that could be construed as a potential conflict of interest.

## Footnotes

1 http://wwwuser.gwdg.de/~evolbio/evolgen/wildmouse/

2 https://github.com/BauerLab/VARSCOT

3 https://bitbucket.org/toolsforpools/pesto/
